# From Principle to Practice: Bridging the Gap in Patient Profiling

**DOI:** 10.1371/journal.pone.0054728

**Published:** 2013-01-25

**Authors:** Jonathan H. Foley, Thomas Orfeo, Anetta Undas, Kelley C. McLean, Ira M. Bernstein, Georges-Etienne Rivard, Kenneth G. Mann, Stephen J. Everse, Kathleen E. Brummel-Ziedins

**Affiliations:** 1 Department of Biochemistry, University of Vermont, Burlington, Vermont, United States of America; 2 Institute of Cardiology, Jagiellonian University School of Medicine, Krakow, Poland; 3 Department of Obstetrics, Gynecology, and Reproductive Sciences, University of Vermont, Burlington, Vermont, United States of America; 4 Department of Hematology-Oncology, Centre Hospitalier Universitaire Sainte-Justine, Montréal, Canada; Maastricht University Medical Center, The Netherlands

## Abstract

The standard clinical coagulation assays, activated partial thromboplastin time (aPTT) and prothrombin time (PT) cannot predict thrombotic or bleeding risk. Since thrombin generation is central to haemorrhage control and when unregulated, is the likely cause of thrombosis, thrombin generation assays (TGA) have gained acceptance as “global assays” of haemostasis. These assays generate an enormous amount of data including four key thrombin parameters (lag time, maximum rate, peak and total thrombin) that may change to varying degrees over time in longitudinal studies. Currently, each thrombin parameter is averaged and presented individually in a table, bar graph or box plot; no method exists to visualize comprehensive thrombin generation data over time. To address this need, we have created a method that visualizes all four thrombin parameters simultaneously and can be animated to evaluate how thrombin generation changes over time. This method uses all thrombin parameters to intrinsically rank individuals based on their haemostatic status. The thrombin generation parameters can be derived empirically using TGA or simulated using computational models (CM). To establish the utility and diverse applicability of our method we demonstrate how warfarin therapy (CM), factor VIII prophylaxis for haemophilia A (CM), and pregnancy (TGA) affects thrombin generation over time. The method is especially suited to evaluate an individual's thrombotic and bleeding risk during “normal” processes (e.g pregnancy or aging) or during therapeutic challenges to the haemostatic system. Ultimately, our method is designed to visualize individualized patient profiles which are becoming evermore important as personalized medicine strategies become routine clinical practice.

## Introduction

Thrombin generation is central to haemorrhage control and when unregulated, is the most likely cause of thrombosis. The dynamic roles of thrombin include procoagulant [1], [2], anticoagulant [3], fibrinolytic [4], [5], mitogenic and motogenic [6], [7] processes. Since many functions of thrombin regulate or directly cause clot formation, its generation is considered a good marker of global haemostasis. For decades the most widely used coagulation assays have been the activated partial thromboplastin time (aPTT) and the prothrombin time (PT). These assays have been invaluable in detecting gross abnormalities in the coagulation system such as factor (f)VIII or fIX deficiency (haemophilias A or B, respectively) or monitoring heparin (reviewed in [8]) or warfarin anticoagulation therapies [9], [10] but have been less useful in predicting thrombotic risk [8] or clinical bleeding phenotype of the haemophilias [11], [12]. Pioneering work by Hemker and colleagues [13], our group [14], and others [12], [15] have demonstrated the vast majority of thrombin is generated well after the plasma (or blood) clot time which is the traditional end point for the aPTT and PT assays. In recent years, thrombin generation assays, thromboelastography and waveform analysis have gained in popularity and become accepted as useful tools to measure “global haemostasis” [16]. Unfortunately, standardization of global assays remains a challenge and has hindered their implementation into clinical practice.

Computational models based upon an individual's coagulant factor composition have also been utilized to further define an individual's thrombin phenotype [17], and therefore, global haemostatic potential. An individual's procoagulant and anticoagulant factor levels act together to generate a unique coagulation phenotype [18], which is represented by their thrombin generation capacity, and like its empirical counterparts, has the potential to identify underlying risk for disease progression. Previously, our group demonstrated, using empirical plasma composition and computational models that the theoretical normal range of thrombin generation varies significantly among healthy individuals with physiologically normal factor levels [19]. This study confirmed on a large scale what small observational studies have shown in the past: that each phase of thrombin generation (i.e. initiation, propagation and termination) is largely regulated by a single or a few coagulation factors. Variation in the tissue factor pathway inhibitor (TFPI) concentration, for instance, has a large effect on the lag time (or clot time/time to 2 nM thrombin) [19], [20], [21] whereas the formation rate and maximum level of thrombin is sensitive to the concentration of factors (fVIII, fIX) which comprise the intrinsic tenase complex [19], [22]. Clinically, thrombin generation parameters such as peak thrombin and total thrombin/endogenous thrombin potential have proved useful in predicting venous thrombosis [23], [24] in at risk individuals and correlating global haemostasis to the bleeding phenotype among patients with hemophilia [12].

Each global assay system, whether empirical or computational, generates multiple outputs and consequently, wide spread use and acceptance of these assays have practical limitations with respect to data presentation. As new assays and technologies emerge and ever increasing amounts of data are collected, it is imperative that data analysis tools evolve to ensure that the data can be presented in a clear, concise and informative manner.

In this paper, we present a novel method to visualize multiple parameters over time. Here, we visualize four thrombin parameters simultaneously and show how each of these parameters changes over time for a given individual in response to a therapeutic intervention or during normal processes associated with haemostatic challenge. Three populations, representing those at risk of haemorrhage (haemophilia A, n = 44), thrombosis (atrial fibrillation, n = 20) or both (pregnancy, n = 20) are evaluated using our method. Thrombin generation for each population was measured differently, and therefore illustrates the utility of our integrating methodology. These diverse populations show dynamic changes in individual thrombin generation profiles over time and thus are ideal for evaluating and validating our data presentation technique.

## Methods

### Ethics statement

Atrial fibrillation patients were recruited and enrolled by Dr. A Undas and advised according to a protocol approved by the Jagiellonian University Ethical Committee (Kraków, Poland). Haemophilia patients were recruited and enrolled by Dr. G-E Rivard and advised according to a protocol approved by the Institutional Review Boards at the Centre Hospitalier Universitaire Sainte-Justine (Montréal, QC) and by the University of Vermont Committees on Human Research (Burlington, VT). Women who intended conception were enrolled and advised according to a protocol approved by the University of Vermont Committees on Human Research. Informed written consent was obtained from all individuals.

### Simulated thrombin generation

For each unique plasma sample, the time course of thrombin generation was simulated using two empirically validated mathematical models termed the “Base model” [25], [26] and the “Protein C model” [27]. In principle, the models differ in their ability to represent the anticoagulant properties of the vasculature. In this regard, the “Base model” describes extravascular coagulation whereas the “Protein C model” describes the coagulation response in the context of the inhibitory potential derived from the vascular endothelium. Both models are built around a series of ordinary differential equations which make use of rate constants derived from experimental measurements made under conditions of saturating concentrations of phospholipids [25]. The “Base model” makes use of the following inputs: empirically determined active concentrations of fII, fV, fVII/fVIIa, fVIII, fIX, fX and the anticoagulants TFPI and antithrombin (AT). The “Protein C model” uses all inputs from the “Base model” as well as the empirically determined active protein C concentration and nominal concentrations of thrombomodulin (TM) [28], an essential anticoagulant cofactor found on the vascular endothelium, which can be altered to represent the amount of TM potentially found in various vessels [29], [30]. Therefore, the minimal information required to simulate thrombin generation is the plasma concentrations of fII, fV, fVII/fVIIa, fVIII, fIX, fX, TFPI and AT (and protein C for the “Protein C” model). For both models, the starting concentration of fVIIa was set to 1% of the starting fVII concentration. The models are initiated by exposing the inputs to 0.5 pM tissue factor for haemophilia simulations (Base model only) or 5 pM tissue factor for warfarin anticoagulation simulations (Base and Protein C models). Using this approach, the concentration versus time profiles for all reactants, including thrombin are determined. Thrombin generation parameters such as the lag time (time to 2 nM thrombin), the maximum rate of thrombin generation (max rate), peak thrombin and total thrombin (area under the thrombin generation profile) can be determined from the time course of thrombin generation [31].

### Empirical thrombin generation

Thrombin generation assays were performed as previously described [32], [33]. Briefly, a 20 µL solution containing 2.5 mM of the thrombin substrate, Z-GGR-AMC and 0.1 M CaCl_2_ was incubated with 80 µL of citrated plasma containing 0.1 mg/mL corn trypsin inhibitor for 3 minutes at 37°C. After this incubation period, thrombin generation was initiated by the addition of 20 µL of relipidated TF (5 pM final) and PCPS (20 µM final) in HEPES buffered saline. As thrombin cleaves Z-GGR-AMC there is an increase in fluorescence which can be used with a series of thrombin standards to calculate the amount of thrombin formed over time in plasma. Using this experimental system, thrombin generation was monitored continuously using a Synergy4 plate reader (BioTek, Winooski, VT, USA). Thrombin generation parameters such as the lag phase, the maximal rate, peak thrombin and total thrombin can be determined from the empirically generated thrombin generation plot.

### Atrial fibrillation population

Patients with diagnosed atrial fibrillation (detailed patient characteristics can be found in Table 1; n = 20; 10 male and 10 female aged 59±6.25 years) varied substantially with respect to their individual risk factors for stroke. Blood was collected from the enrolled patients on 6 occasions during the study period and used to make citrated platelet poor plasma which was aliquoted and stored at −80°C. The first sample was collected just prior to starting warfarin therapy. Subsequent samples were collected on days 3, 5, 7, 14 and 30 after initiating warfarin therapy. On each day, each subjects' plasma composition was determined (6 days×20 subjects = 120 unique plasma compositions) primarily by using routine activity-based clinical clotting assays [34]. The concentrations of fII, fV, fVII/fVIIa, fVIII, fIX, fX and the anticoagulants TFPI and AT were used to simulate thrombin generation using the “Base model” and “Protein C model”.

**Table 1:** Atrial fibrillation patient characteristics.

Patient	Sex	Age	CAD	HT	DB	SM	HC	ST	ASA	ACEI	STAT	HF	BMI
1	M	68	0	1	1	0	1	1	1	1	1	1	35
2	F	59	0	0	0	0	0	0	0	0	0	0	21
3	M	66	0	1	0	1	1	0	0	1	1	0	27
4	F	58	1	0	1	0	0	1	1	0	0	1	34
5	M	59	0	1	0	1	1	0	0	1	1	1	28
6	F	54	0	1	0	0	0	0	0	1	0	0	28
7	F	52	0	1	0	1	0	0	0	1	0	0	29
8	F	60	0	0	0	0	0	0	0	0	0	0	21
9	F	63	0	1	1	0	1	0	0	1	1	1	28
10	M	68	0	1	0	0	0	0	0	1	0	0	29
11	F	59	0	1	0	0	1	0	0	1	1	0	24
12	F	51	0	1	0	0	1	0	0	1	1	0	34
13	M	48	0	1	0	0	1	0	0	1	1	0	31
14	M	69	0	1	0	1	0	0	0	1	0	0	31
15	M	51	1	0	0	1	0	0	1	0	0	0	28
16	M	59	0	1	0	0	1	0	0	1	1	0	24
17	F	53	0	1	0	0	1	0	0	1	1	1	30
18	M	61	0	0	0	0	0	0	0	0	0	0	27
19	M	64	0	0	0	0	1	0	0	0	1	1	25
20	F	54	1	1	0	1	1	1	1	0	1	1	31

Yes = 1.

No = 0.

CAD: Coronary artery disease.

HT: Hypertension.

DB: Diabetes.

SM: Smoker.

HC: Hypercholesteremia.

ST: Stroke/transient ischemic attack.

ASA: Aspirin.

ACEI: ACE inhibitors.

STAT: Statins.

HF: Heart failure.

BMI: Body mass index.

### Haemophilia population

Patients with clinically severe haemophilia A had fVIII:C <1% at the time of diagnosis (age range 16–33) [35]. Each subjects' plasma composition was determined primarily by using routine activity-based clinical clotting assays. The concentrations of fII, fV, fVII/fVIIa, fVIII, fIX, fX and the anticoagulants TFPI and AT were used as measured to simulate thrombin generation using the “Base model”. Since all subjects have clinically severe haemophilia A (fVIII <1%) and their fVIII levels varied significantly at the time of blood collection, the fVIII concentration was electronically set at 100% at time zero (baseline). The thrombin generating capacity was followed over 7 half-lives (42–168 hours; t_1/2_ = 6–24 hours) of fVIII to demonstrate the theoretical fluctuations in thrombin generating capacity during the course of fVIII prophylaxis.

### Pregnant population

Women who intended conception were enrolled in the initial study [36]. Study participants (aged 18–40 years) were healthy non-smokers with no history of hypertension, diabetes mellitus, autoimmune disease or haemostatic disorders. At the time of enrollment, all women had regular menstrual cycles (n = 20 pregnant; n = 10 non-pregnant controls). Blood was collected from enrolled patients up to 4 times during the study. Blood was centrifuged immediately to produce citrated platelet poor plasma which was subsequently aliquotted and stored at −80°C. Pre-pregnancy samples were collected during the follicular phase of the menstrual cycle. Early and late pregnancy samples were collected at 11–15 menstrual weeks and 30–34 weeks, respectively. Post-pregnancy samples were collected after breastfeeding ceased which was between 6 and 24 months after delivery in all cases. Post-pregnancy samples were also collected in the follicular phase of the menstrual cycle. Enrolled women who did not become pregnant remained in the study as control subjects (data not shown). These women provided blood samples pre-pregnancy and approximately 2.5 years after the initial blood draw. The thrombin generation capacities of these women were previously reported by McLean *et al*. [33].

### Dynamic visualization of thrombin generation

Thrombin generation parameters were determined either computationally or empirically as described in the “*Simulated/Empirical thrombin generation*” sections of the *Online Methods*. Thrombin parameters depicting the kinetics of warfarin anticoagulation or the net result of decreasing fVIII during prophylaxis in haemophilia A were generated using the computational models. Thrombin parameters depicting global haemostatic changes during pregnancy were determined empirically. For each individual, the lag time (time to 2 nM thrombin), maximal rate of thrombin generation, peak thrombin and total thrombin (area under the curve) were plotted against time using the motion chart gadget which is available in Google Docs (Mountain View, CA) spreadsheets. Using this gadget, 5 dimensional plots were created. In these plots, the lag time is depicted on the y-axis, maximal rate of thrombin generation is depicted on the x-axis, peak thrombin is represented by the colour, and total thrombin is represented by the relative size of each data point [19]. A large, red circle in the lower right quadrant is representative of a high thrombin generating capacity whereas a small, blue circle in the upper left quadrant represents a low thrombin generating capacity. The time component is shown by animating each point to move as thrombin generation parameters change over time. Videos depicting changes in thrombin generation over time were captured using Screenflow software (Nevada City, CA). Labels were added to videos using Final Cut Pro software (Apple, Inc., Cupertino, CA). Each figure was created by taking screen captures of relevant videos.

## Results

### Simulated thrombin generation during warfarin therapy in atrial fibrillation

**Figure 1:**
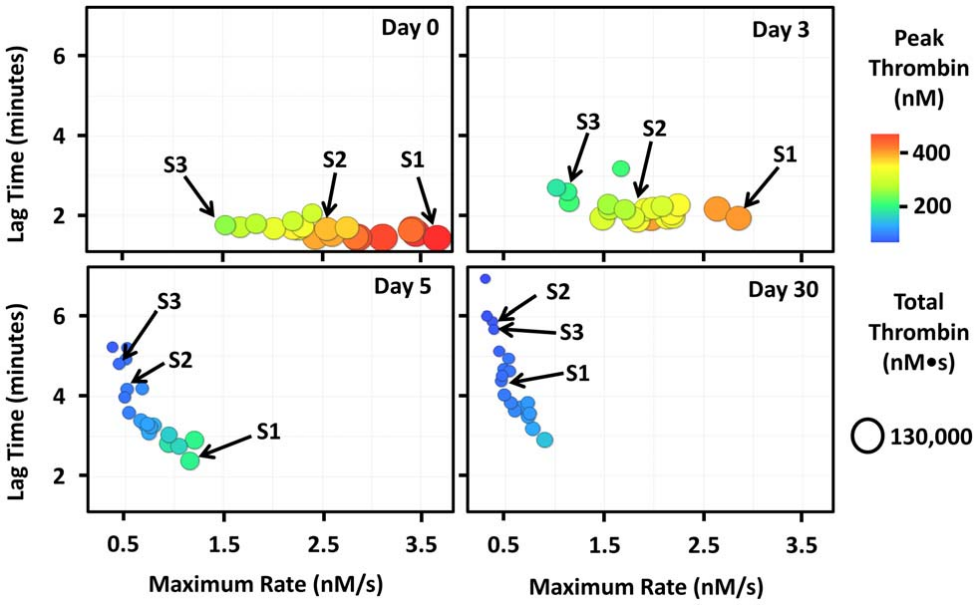
The kinetics of warfarin anticoagulation in patients with atrial fibrillation. Thrombin generating capacity was simulated by inputting each subjects' factor composition into our mathematical model. Each point (circle) in the figure is representative of a single individual's thrombin generating capacity before and during warfarin therapy. A video showing the dynamic thrombin generating capacity over time can be viewed from Movie S1. All subjects, including the 3 highlighted (S1: subject 1, S2: subject 2 and S3: subject 3), show a time dependent reduction in thrombin generating capacity (marginally increased lag time, decreased maximal rate, decreased peak and total thrombin) in response to warfarin therapy. Note that the peak thrombin scale ranges from 0–500 nM.

Patients with atrial fibrillation were enrolled and provided us with plasma samples just prior to commencing warfarin therapy (day 0) and on days 3, 5, 7, 14 and 30 of warfarin therapy. The coagulation factor composition for each unique plasma sample was used to simulate the time course of thrombin generation using two empirically validated mathematical models termed the “Base model” and the “Protein C model”. The mechanism of warfarin anticoagulation is well-established (reviewed in [37]) and the associated dynamic reduction in thrombin generating capacity over time can be visualized in Movie S1. These data which depict thrombin generation parameters derived from the “Base model” are consistent with previous reports (reviewed in [37]). Figure 1 was created by taking screenshots of Movie S1 over time. Figure 1 shows that all subjects, including the 3 highlighted (S1, S2 and S3), have a reduced thrombin generating capacity in response to warfarin therapy. After 3 days on warfarin, subjects S1, S2 and S3 have reduced peak and total thrombin and a reduced maximal rate of thrombin generation compared to baseline. In addition, each subject has a slightly prolonged lag time. This trend continues through day 5 where S2 and S3 are approaching a stable thrombin generating capacity suggesting stable anticoagulation. By day 30, all 3 subjects are stably anticoagulated which is implied by their consistent but drastically reduced thrombin generating capacity.

**Figure 2:**
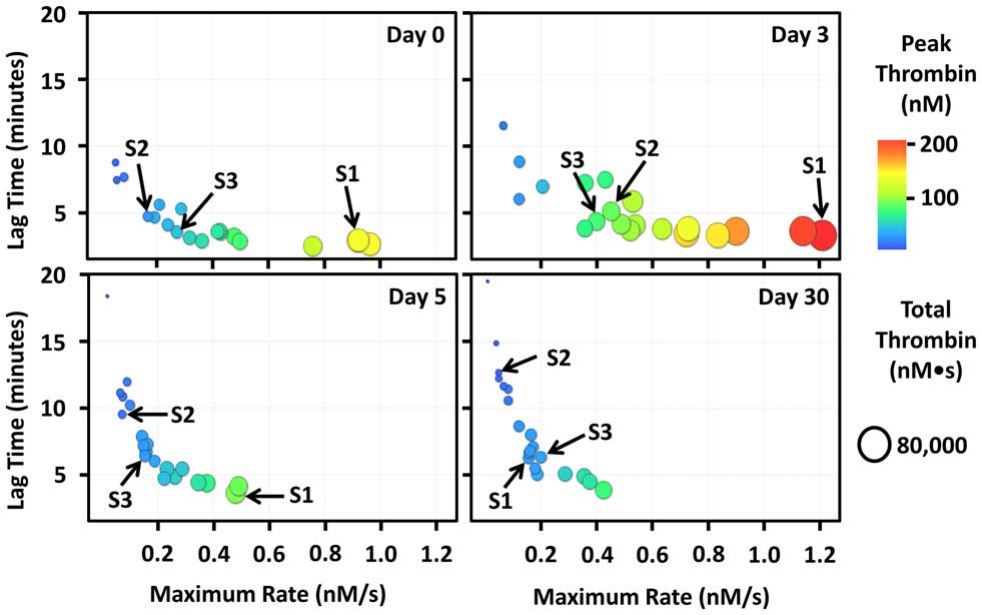
The effect of the protein C pathway on the kinetics of Warfarin anticoagulation in patients with atrial fibrillation. Thrombin generating capacity was simulated by inputting each subjects' factor composition into our mathematical model containing the protein C pathway. Each point (circle) in the figure is representative of a single individual's thrombin generating capacity before and during warfarin therapy. A video showing the dynamic thrombin generating capacity over time can be viewed from the Movie S2. All subjects show a time dependent reduction in thrombin generating capacity (increased lag time, increased maximal rate, decreased peak and total thrombin) in response to warfarin therapy. Most subjects, including the subjects highlighted (S1: subject 1, S2: subject 2 and S3: subject 3), have an increased maximal rate, peak and total thrombin and a marginally increased lag time 3 days after starting warfarin therapy. After day 3, every subjects' thrombin generating capacity decreases in a similar fashion to that shown using our “Base model” (figure 1). Note that the peak thrombin scale ranges from 0–200 nM.

Using a similar approach to that employed in the creation of Movie S1, thrombin generation data displayed in Movie S2 was generated based on a mathematical simulation that incorporated the effect of the protein C pathway whereas the previous model did not. Figure 2 was created by taking screenshots of Movie S2 over time. As with the Base model (presented in Movie S1 and Figure 1), all subjects, including those highlighted (S1, S2 and S3; same as highlighted in Figure 1) become stably anticoagulated as a result of warfarin therapy. The key difference occurs after 3 days on warfarin: most subjects including S1, S2 and S3 paradoxically have an increased thrombin generating capacity compared to baseline. Our simulations suggest that peak and total thrombin and the maximal rate of thrombin generation increases during the initial phase of warfarin therapy. After 3 days on warfarin, the lag time remains constant for all three highlighted subjects as it does for >75% of the other subjects. After 5 days on warfarin all 3 highlighted subjects have a reduced thrombin generating capacity and subject S2 and S3 become stably anticoagulated. By day 30 all subjects are stably anticoagulated.

### Simulated thrombin generation during fVIII prophylaxis in haemophilia A

**Figure 3:**
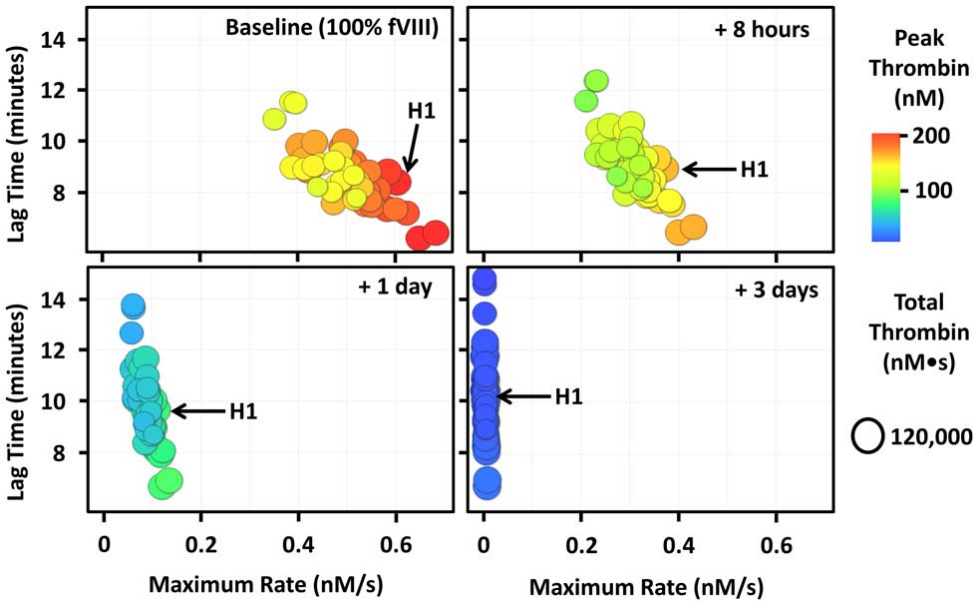
Dynamic reduction of thrombin generation parameters over time in a severe haemophilia A population. Thrombin generating capacity was simulated by inputting each subject's factor composition into our mathematical model. Each point (circle) in the figure is representative of a single individual's thrombin generating capacity. A video showing the effects of decaying fVIII on the dynamic thrombin generating capacity can be viewed from the Movie S3. Since each subject has clinically severe haemophilia A (fVIII <1%), the fVIII concentration was set at 100% at time zero (baseline). The thrombin generating capacity was followed over 7 half-lives of fVIII (t_1/2_ = 12.2 hours) which represents the approximate time between prophylactic fVIII doses. All individuals, including subject H1, showed a decrease in thrombin generating capacity (decreased maximal rate and peak thrombin and marginally decreased total thrombin and marginally increased lag time) as fVIII decayed. Note that the peak thrombin scale ranges from 0–200 nM.

Patients with severe haemophilia were enrolled and provided us with plasma samples which were used to determine their factor composition. The coagulation factor composition for each unique plasma sample was used to simulate the time course of thrombin generation using the empirically validated “Base model”. Since all subjects have clinically severe haemophilia A (fVIII <1%) and their fVIII levels varied significantly at the time of blood collection, the fVIII concentration was set at 100% at time zero (baseline) to reflect the ideal goal of administering fVIII (i.e. to restore fVIII levels to normal). The thrombin generating capacity was followed over 7 half-lives of fVIII (t_1/2_ = 12.2 hours) to demonstrate the theoretical fluctuations in thrombin generating capacity during the course of fVIII prophylaxis. At 100% (“baseline”) fVIII, there is significant individual variation in thrombin generating capacity among individuals with severe haemophilia A (Movie S3) which is consistent with previous work [12], [19]. The maximum rate of thrombin generation ranges from 0.35 to 0.7 nM/s, peak thrombin ranges from 100 to 200 nM and lag time ranges from 7 to 12 minutes. Figure 3 was created by taking screenshots of Movie S3 over time. Using subject H1 as an example, it is evident that maximal rate of thrombin generation and peak thrombin levels decrease as time passes and fVIII decays. The lag time and total thrombin levels are affected less in this tissue factor stimulated model of coagulation and thrombin generation.

**Figure 4:**
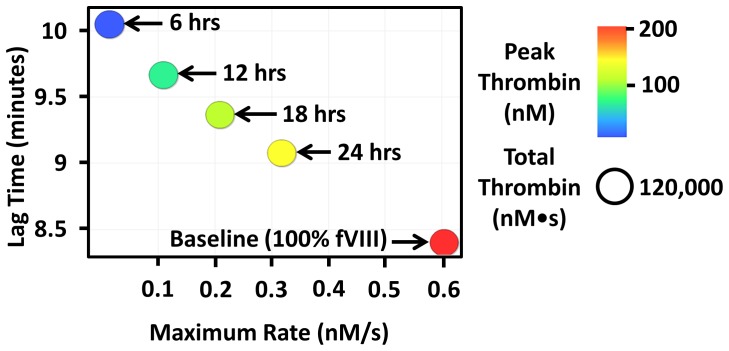
The effect of factor VIII product half-life on the dynamics of thrombin generation over time 32 hours post administration of fVIII. Thrombin generating capacity was simulated by inputting subject H1's factor levels into our mathematical model. A video demonstrating the relative extension between doses when the half-life of fVIII is increased is provided in the Movie S4. The thrombin generation capacity is also shown at 32 hours for 4 hypothetical fVIII products with half-lives of 6, 12, 18 and 24 hours. The baseline (100% fVIII) thrombin generating capacity at time zero is shown as a reference. By 32 hours, the 6 hour product has decayed to ∼1% which coincides with the approximate timing between prophylactic doses of fVIII. Note that the peak thrombin scale ranges from 0–200 nM.

To show the effect of increased fVIII product half-life on thrombin generating capacity, thrombin parameters were generated using our “Base model” and the coagulation factor levels of subject H1 over 7 half-lives of fVIII. The effect of 4 hypothetical fVIII products on thrombin generation is shown in Movie S4. The products' have half-lives range from 6 (6 hrs) to 24 hours (24 hrs). As time passes, fVIII decays and the thrombin generating capacity of subject H1 decreases. Movie S4 shows that fVIII products with a longer half-life maintain a relatively higher thrombin generating capacity for a longer period than the shorter half-life products. In the video, once a given fVIII product level falls below 1%, the plot disappears. The time to disappearance for each fVIII product represents the approximate relative time between fVIII doses. Figure 4 was created by taking a screenshot of Movie S4 32 hours after electronic “infusion” of fVIII. Figure 4 depicts baseline thrombin generating capacity just after fVIII infusion (i.e. 100% fVIII) for subject H1 and the thrombin generating capacity expected after 32 hours with the 4 hypothetical fVIII products. The 32 hour time point represents the time required for the 6 hour fVIII product to fall to 1% which, based on modern prophylactic regiments, is when an additional dose of (6 hour) fVIII would be required [38]. By definition the 12, 18 and 24 hour fVIII products have not decayed as quickly and therefore do not need to be supplemented with an additional dose of fVIII at this time.

### Empirical thrombin generation during pregnancy

**Figure 5:**
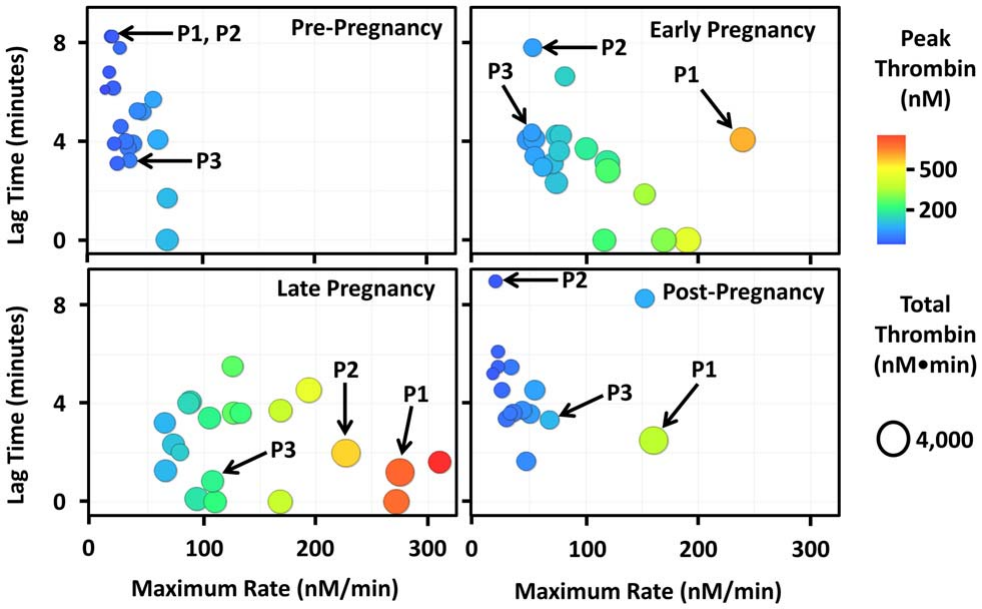
Dynamic thrombin generation during the course of pregnancy. Thrombin generating capacity was determined empirically using a thrombin generation assay. Each point (circle) in the figure is representative of a single individual's thrombin generating capacity. A video showing changes in the dynamic thrombin generating capacity during pregnancy can be viewed from the Movie S5. All subjects, including the 3 highlighted (P1: pregnant subject 1, P2: pregnant subject 2 and P3: pregnant subject 3), have increased thrombin generating capacity (decreased lag time, increased maximal rate, increased peak and total thrombin) in early pregnancy. The thrombin generation capacity increases further in late pregnancy and post-pregnancy returns to near baseline levels for most individuals. Note that the peak thrombin scale ranges from 0–750 nM.

Patients planning pregnancy were enrolled and provided us with plasma samples which were used to empirically measure thrombin generation using a thrombin generation assay. Movie S5 shows that most subjects (16 of 19) have a lag time of between 3 and 8 minutes at baseline (pre-pregnancy). All subjects have a maximum rate of thrombin generation less than 100 nM/min and peak thrombin less than 200 nM. Total thrombin ranges from 745 nM-min to 2675 nM-min in these individuals at baseline. In early pregnancy (11 to 15 weeks), there is a trend toward a procoagulant state with the lag time decreasing, maximum rate of thrombin generation increasing and both peak and total thrombin increasing. In late pregnancy (30 to 34 weeks), there is a further reduction in the lag time. The maximum rate of thrombin generation and peak and total thrombin levels increase further compared to early pregnancy. After pregnancy and after breast feeding has ceased (6 to 24 months after delivery), the thrombin generating capacity returns to the range observed pre-pregnancy. Post-pregnancy, the lag time is between 3 and 8 minutes for most individuals and the maximum rate of thrombin generation is less than 100 nM/min for all but 2 individuals. Peak and total thrombin are also similar to pre-pregnancy values in all but 2 individuals. Figure 5 was created by taking screenshots of Movie S5 over time.

## Discussion

Conventional approaches to data analysis combined with standard statistical methods have been limited in their ability to identify at risk individuals. Our method integrates multiple selected measures characteristic of individual coagulation profiles and provides a unique level of resolving power with respect to differences between individuals including the potential for risk assessment of hemorrhagic and thrombotic events and monitoring of anticoagulation [19]. Our method can be generalized further to take multiple measures from any type of instrument or values from standard clinical tests (i.e. PT, aPTT, etc), and repackage them into an integrated form that allows individuals to be monitored over time and directly compared to other individuals evaluated the same way.

Our method has clear advantages over currently used data presentation techniques which describe thrombin generation parameters. Typically, these values are tabulated and reported as a mean ± standard deviation or graphically with each mean ± standard deviation value presented in a bar graph or box plot. Our method is unique in that it provides a visual representation of all thrombin parameters in a single plot and captures how these parameters change over time in response to clinical events or therapies which alter an individual's haemostatic potential. Making use of three discrete populations with “abnormal” haemostasis we have demonstrated the utility of our method in visualizing changes in thrombin generation during warfarin therapy, fVIII prophylaxis for haemophilia A and pregnancy.

In the current study only one method of determining thrombin generation was used for each population but based on the extensive empirical validation of our mathematical model [25], [26], [27] we expect that simulated and empirical thrombin generation data would be similar. Our video plot (Movie S1) shows that the atrial fibrillation group is stably anticoagulated within 5 days of commencing warfarin therapy. These data, generated using computational methods, are consistent with the well-established role of warfarin in decreasing the production of vitamin K dependent proteins [37] which results in reduced thrombin generation *in vivo* [39], *in vitro* [40] and *in silico* [41]. Adding the protein C pathway to our mathematical model and plotting the simulated data using our video plot method, we have identified a theoretical window in which patients on warfarin may be at an increased risk of thrombosis. Our video plot based on the “Protein C model” (Movie S2) shows that all patients have an increased thrombin generating capacity 3 days after starting warfarin therapy. After day 3, the thrombin generating capacity decreases substantially as each patient becomes stably anticoagulated. This paradoxical and theoretical increase in thrombotic risk can be explained by the relatively short half-life of protein C compared to other vitamin K dependent proteins such as prothrombin and fX [42]. Since protein C levels decrease faster during warfarin therapy than prothrombin and fX, there is a window of time where the anticoagulant pathway afforded by protein C is diminished to a greater extent than that of procoagulant pathways comprising the other vitamin K dependent proteins. Interestingly, an increased thrombin generating capacity on day 3 is only marginally associated with an increased lag time. The lag time is the thrombin parameter which most closely resembles the clot time in the PT assay which is clinically used to monitor warfarin therapy. The simulated lag times are consistent with the insensitivity of the PT assay to protein C levels [43] but nonetheless show a theoretical increase in thrombin generating capacity during the early stages of warfarin therapy. Therefore, modeling the kinetics of warfarin anticoagulation may be useful in identifying individuals who are most at risk of thrombosis during the early stages of warfarin therapy.

We have also illustrated the utility of our method in monitoring thrombin generating capacity among patients with severe haemophilia. Movie S3, generated using simulated thrombin generation data, shows that the maximal rate of thrombin generation and peak thrombin decreases dramatically as fVIII decays while the lag time and total thrombin are only marginally decreased. As reviewed previously [38], the goal in prophylactic factor replacement therapy is to keep the fVIII concentration above 1% to significantly reduce the risk of bleeding. Our video (Movie S4) shows the relative timing of reduced thrombin generating capacity in haemophilia A during prophylactic fVIII replacement therapy and illustrates very clearly the clinical benefit of theoretical fVIII products with a prolonged half-life. Since the pharmacokinetics of fVIII is not known in these patients, we fixed the fVIII concentration at 100% and allowed fVIII to decay with a half-life of 12.2 hours (Figure 3). An additional potential limitation is that the effects of von Willebrand factor levels on the efficacy and half-life of fVIII replacement products is not currently part of the model. Thus, we acknowledge that this does not represent the actual dynamic thrombin generating capacity of the patients enrolled since the fVIII half-live is unlikely to be exactly 12.2 hours, but nonetheless the video demonstrates how thrombin generating capacity changes over the course of fVIII prophylaxis in patients with similar fVIII half-lives.

Finally, using our pregnant population we show that the utility of this method of data presentation is not exclusive to simulated thrombin generation parameters but can also be used to chart thrombin generating capacity using empirical parameters from thrombin generation assays. Consistent with previous reports [44], [45], [46], our pregnant population has an increased procoagulant tendency in early pregnancy which increases further in late pregnancy. After delivery and cessation of breast feeding (post-pregnancy) the video shows that thrombin generating capacity returns to pre-pregnancy levels. The plot also very clearly identifies subjects who contain an endogenous activator within their plasma (lag time = 0 minutes). Using a previously described assay [47], it was determined that these subjects had endogenous fIXa or fXIa activity [48].

The marriage between simulated thrombin generation and our method allows for rapid identification of individuals with abnormal thrombin generation kinetics. In recent years, considerable effort and resources have been devoted to the development of personalized medicine, but many hurdles remain [49]. Any tool which simplifies the identification of at risk individuals will likely streamline the implementation of personalized therapies, thus improving patient care and outcomes. The ways that the general population and scientific community consume and use data have changed drastically over the past few years. As recently as 5 years ago the utility of our method would have been limited to a desktop computer. Today, however, the ubiquity of the internet combined with advances in computing power make this method accessible via desktop computers as well as tablets and smartphones.

## Supporting Information

Movie S1 The kinetics of warfarin anticoagulation in patients with atrial fibrillation. Each unit of time (spanning 1900–1930) in the video represents 1 day. Thrombin generating capacity was simulated by inputting each subject's factor composition into our mathematical model. Each point (circle) in the figure is representative of a single individual's thrombin generating capacity before and during warfarin therapy. Each patient (n = 20) is represented by a circle. The red arrow and accompanying circle is representative of a theoretical individual with 100% of all coagulation factors.
(MOV)

Movie S2 The effect of the protein C pathway on the kinetics of Warfarin anticoagulation in patients with atrial fibrillation. Each unit of time (spanning 1900–1930) in the video represents 1 day. Thrombin generating capacity was simulated by inputting each subject's factor composition into our mathematical model containing the protein C pathway. Each point (circle) in the figure is representative of a single individual's thrombin generating capacity before and during warfarin therapy. The red arrow and accompanying circle is representative of a theoretical individual with 100% of all coagulation factors.
(MOV)

Movie S3 Dynamic reduction of thrombin generation parameters over time in a severe haemophilia A population. The fVIII product used here has a half-life of 12.2 hours. Each unit of time (spanning 1900–1912) in the video represents 8 hours. Thrombin generating capacity was simulated by inputting each subject's factor composition into our mathematical model. Each point (circle) in the figure is representative of a single individual's thrombin generating capacity. The red arrow and accompanying circle is representative of a theoretical individual with 100% of all coagulation factors.
(MOV)

Movie S4 The effect of factor VIII product half-life on the dynamics of thrombin generation over time post administration of fVIII. Each unit of time (spanning 1900–1921) in the video represents 8 hours. Thrombin generating capacity was simulated by inputting a single subject's (H1 from Figure 4) factor levels into our mathematical model. The theoretical fVIII products used here have half-lives of 6, 12.2, 18 and 24 hours. By definition the 6 hour product decays much faster than products with longer half-lives and therefore the thrombin generating capacity of the 6 hour product declines fastest followed by the 12.2, 18 and 24 hour products.
(MOV)

Movie S5 Dynamic thrombin generation during the course of pregnancy. Each unit of time (spanning 1900–1904) in the video is representative of a single phase of pregnancy (in order pre-pregnancy, early pregnancy, late pregnancy and post pregnancy). Thrombin generating capacity was determined empirically using a thrombin generation assay. Each point (circle) in the figure is representative of a single individual's thrombin generating capacity. The red arrow and accompanying circle is representative of the mean thrombin generating capacity of the group at the pre-pregnancy time point.
(MOV)

## References

[1] Nesheim ME, Taswell JB, Mann KG (1979) The contribution of bovine Factor V and Factor Va to the activity of prothrombinase. J Biol Chem 254: 10952–10962. 500617

[2] Butenas S, Branda RF, van't Veer C, Cawthern KM, Mann KG (2001) Platelets and phospholipids in tissue factor-initiated thrombin generation. Thromb Haemost 86: 660–667. 11522019

[3] Kisiel W, Canfield WM, Ericsson LH, Davie EW (1977) Anticoagulant properties of bovine plasma protein C following activation by thrombin. Biochemistry 16: 5824–5831. 588557 10.1021/bi00645a029

[4] Shatos MA, Orfeo T, Doherty JM, Penar PL, Collen D, et al (1995) Alpha-thrombin stimulates urokinase production and DNA synthesis in cultured human cerebral microvascular endothelial cells. Arterioscler Thromb Vasc Biol 15: 903–911. 7600122 10.1161/01.atv.15.7.903

[5] Levin EG, Marzec U, Anderson J, Harker LA (1984) Thrombin stimulates tissue plasminogen activator release from cultured human endothelial cells. J Clin Invest 74: 1988–1995. 6542570 10.1172/JCI111620 PMC425386

[6] Belloni PN, Carney DH, Nicolson GL (1992) Organ-derived microvessel endothelial cells exhibit differential responsiveness to thrombin and other growth factors. Microvasc Res 43: 20–45. 1608338 10.1016/0026-2862(92)90004-9

[7] Herbert JM, Dupuy E, Laplace MC, Zini JM, Bar Shavit R, et al (1994) Thrombin induces endothelial cell growth via both a proteolytic and a non-proteolytic pathway. Biochem J 303 Pt 1: 227–231 7945245 10.1042/bj3030227 PMC1137580

[8] Eikelboom JW, Hirsh J (2006) Monitoring unfractionated heparin with the aPTT: time for a fresh look. Thromb Haemost 96: 547–552. 17080209

[9] Quick AJ, Stanley-Brown M, Bancroft FW (1935) A study of the coagulation defect in hemophilia and in jaundice. Am J Med Sci 190: 501–511.

[10] Adcock DM, Duff S (2000) Enhanced standardization of the International Normalized Ratio through the use of plasma calibrants: a concise review. Blood Coagul Fibrinolysis 11: 583–590. 11085277 10.1097/00001721-200010000-00001

[11] Siegemund T, Petros S, Siegemund A, Scholz U, Engelmann L (2003) Thrombin generation in severe haemophilia A and B: the endogenous thrombin potential in platelet-rich plasma. Thromb Haemost 90: 781–786. 14597971 10.1160/TH03-01-0027

[12] Dargaud Y, Beguin S, Lienhart A, Al Dieri R, Trzeciak C, et al (2005) Evaluation of thrombin generating capacity in plasma from patients with haemophilia A and B. Thromb Haemost 93: 475–480. 15735797 10.1160/TH04-10-0706

[13] Hemker HC, Al Dieri R, De Smedt E, Beguin S (2006) Thrombin generation, a function test of the haemostatic-thrombotic system. Thromb Haemost 96: 553–561. 17080210

[14] Mann KG, Brummel K, Butenas S (2003) What is all that thrombin for? J Thromb Haemost 1: 1504–1514. 12871286 10.1046/j.1538-7836.2003.00298.x

[15] Hron G, Kollars M, Binder BR, Eichinger S, Kyrle PA (2006) Identification of patients at low risk for recurrent venous thromboembolism by measuring thrombin generation. JAMA 296: 397–402. 16868297 10.1001/jama.296.4.397

[16] Nair SC, Dargaud Y, Chitlur M, Srivastava A (2010) Tests of global haemostasis and their applications in bleeding disorders. Haemophilia 16 Suppl 5: 85–92. 20590862 10.1111/j.1365-2516.2010.02304.x

[17] Brummel-Ziedins KE, Pouliot RL, Mann KG (2004) Thrombin generation: phenotypic quantitation. J Thromb Haemost 2: 281–288. 14995991 10.1046/j.1538-7933.2003.00576.x

[18] Brummel-Ziedins KE, Orfeo T, Rosendaal FR, Undas A, Rivard GE, et al (2009) Empirical and theoretical phenotypic discrimination. J Thromb Haemost 7 Suppl 1: 181–186. 19630796 10.1111/j.1538-7836.2009.03426.x PMC3395063

[19] Danforth CM, Orfeo T, Everse SJ, Mann KG, Brummel-Ziedins KE (2012) Defining the boundaries of normal thrombin generation: investigations into hemostasis. PLoS One 7: e30385. 22319567 10.1371/journal.pone.0030385 PMC3271084

[20] Brodin E, Appelbom H, Osterud B, Hilden I, Petersen LC, et al (2009) Regulation of thrombin generation by TFPI in plasma without and with heparin. Transl Res 153: 124–131. 19218095 10.1016/j.trsl.2008.12.004

[21] van 't Veer C, Mann KG (1997) Regulation of tissue factor initiated thrombin generation by the stoichiometric inhibitors tissue factor pathway inhibitor, antithrombin-III, and heparin cofactor-II. J Biol Chem 272: 4367–4377. 9020158 10.1074/jbc.272.7.4367

[22] Allen GA, Wolberg AS, Oliver JA, Hoffman M, Roberts HR, et al (2004) Impact of procoagulant concentration on rate, peak and total thrombin generation in a model system. J Thromb Haemost 2: 402–413. 15009455 10.1111/j.1538-7933.2003.00617.x

[23] Lutsey PL, Folsom AR, Heckbert SR, Cushman M (2009) Peak thrombin generation and subsequent venous thromboembolism: the Longitudinal Investigation of Thromboembolism Etiology (LITE) study. J Thromb Haemost 7: 1639–1648. 19656279 10.1111/j.1538-7836.2009.03561.x PMC2763356

[24] Besser M, Baglin C, Luddington R, van Hylckama Vlieg A, Baglin T (2008) High rate of unprovoked recurrent venous thrombosis is associated with high thrombin-generating potential in a prospective cohort study. J Thromb Haemost 6: 1720–1725. 18680535 10.1111/j.1538-7836.2008.03117.x

[25] Hockin MF, Jones KC, Everse SJ, Mann KG (2002) A model for the stoichiometric regulation of blood coagulation. J Biol Chem 277: 18322–18333. 11893748 10.1074/jbc.M201173200

[26] Butenas S, Orfeo T, Gissel MT, Brummel KE, Mann KG (2004) The significance of circulating factor IXa in blood. J Biol Chem 279: 22875–22882. 15039440 10.1074/jbc.M400531200

[27] Bravo MC, Orfeo T, Mann KG, Everse SJ (2012) Modeling of human factor Va inactivation by activated protein C. BMC Syst Biol 6: 45. 22607732 10.1186/1752-0509-6-45 PMC3403913

[28] Brummel-Ziedins KE, Orfeo T, Callas PW, Gissel M, Mann KG, et al (2012) The prothrombotic phenotypes in familial protein C deficiency are differentiated by computational modeling of thrombin generation. PLoS One 7: e44378. 22984498 10.1371/journal.pone.0044378 PMC3440432

[29] Busch C, Cancilla PA, DeBault LE, Goldsmith JC, Owen WG (1982) Use of endothelium cultured on microcarriers as a model for the microcirculation. Lab Invest 47: 498–504. 6752573

[30] Mann KG (2011) Thrombin generation in hemorrhage control and vascular occlusion. Circulation 124: 225–235. 21747067 10.1161/CIRCULATIONAHA.110.952648 PMC3138077

[31] Brummel-Ziedins K, Vossen CY, Rosendaal FR, Umezaki K, Mann KG (2005) The plasma hemostatic proteome: thrombin generation in healthy individuals. J Thromb Haemost 3: 1472–1481. 15978105 10.1111/j.1538-7836.2005.01249.x PMC1414093

[32] Hemker HC, Giesen P, AlDieri R, Regnault V, de Smed E, et al (2002) The calibrated automated thrombogram (CAT): a universal routine test for hyper- and hypocoagulability. Pathophysiol Haemost Thromb 32: 249–253. 13679651 10.1159/000073575

[33] McLean KC, Bernstein IM, Brummel-Ziedins KE (2012) Tissue factor-dependent thrombin generation across pregnancy. Am J Obstet Gynecol 207: 135 e131–136. 22840726 10.1016/j.ajog.2012.05.027 PMC3661010

[34] Brummel-Ziedins K, Undas A, Orfeo T, Gissel M, Butenas S, et al (2008) Thrombin generation in acute coronary syndrome and stable coronary artery disease: dependence on plasma factor composition. J Thromb Haemost 6: 104–110. 17944993 10.1111/j.1538-7836.2007.02799.x

[35] Gissel M, Whelihan MF, Ferris LA, Mann KG, Rivard GE, et al (2012) The influence of prophylactic factor VIII in severe haemophilia A. Haemophilia 18: 193–199. 21899664 10.1111/j.1365-2516.2011.02638.x PMC3778653

[36] Hale SA, Schonberg A, Badger GJ, Bernstein IM (2009) Relationship between prepregnancy and early pregnancy uterine blood flow and resistance index. Reprod Sci 16: 1091–1096. 19657141 10.1177/1933719109341843 PMC2882858

[37] Hirsh J, Dalen J, Anderson DR, Poller L, Bussey H, et al (2001) Oral anticoagulants: mechanism of action, clinical effectiveness, and optimal therapeutic range. Chest 119: 8S–21S. 11157640 10.1378/chest.119.1_suppl.8s

[38] Manco-Johnson M (2007) Comparing prophylaxis with episodic treatment in haemophilia A: implications for clinical practice. Haemophilia 13 Suppl 2: 4–9. 10.1111/j.1365-2516.2007.01499.x 17685917

[39] Conway EM, Bauer KA, Barzegar S, Rosenberg RD (1987) Suppression of hemostatic system activation by oral anticoagulants in the blood of patients with thrombotic diatheses. J Clin Invest 80: 1535–1544. 3680513 10.1172/JCI113239 PMC442421

[40] Dargaud Y, Desmurs-Clavel H, Marin S, Bordet JC, Poplavsky JL, et al (2008) Comparison of the capacities of two prothrombin complex concentrates to restore thrombin generation in plasma from orally anticoagulated patients: an in vitro study. J Thromb Haemost 6: 962–968. 18373620 10.1111/j.1538-7836.2008.02964.x

[41] Orfeo T, Gissel M, Butenas S, Undas A, Brummel-Ziedins KE, et al (2011) Anticoagulants and the propagation phase of thrombin generation. PLoS One 6: e27852. 22125631 10.1371/journal.pone.0027852 PMC3220702

[42] Brummel-Ziedins K, Orfeo T, Jenny NS, Everse SJ, Mann KG (2009) Blood Coagulation and Fibrinolysis. In: Greer JP, Foerster J, Rodgers GM, Paraskevas F, Glader B et al.., editors. Wintrobe's Clinical Hematology. Philadelphia: Wolters Kluwer, Lippincott Williams & Wilkins. pp. 528–619.

[43] Khor B, Van Cott EM (2010) Laboratory tests for protein C deficiency. Am J Hematol 85: 440–442. 20309856 10.1002/ajh.21679

[44] Eichinger S, Weltermann A, Philipp K, Hafner E, Kaider A, et al (1999) Prospective evaluation of hemostatic system activation and thrombin potential in healthy pregnant women with and without factor V Leiden. Thromb Haemost 82: 1232–1236. 10544904

[45] Dargaud Y, Hierso S, Rugeri L, Battie C, Gaucherand P, et al (2010) Endogenous thrombin potential, prothrombin fragment 1+2 and D-dimers during pregnancy. Thromb Haemost 103: 469–471. 20024504 10.1160/TH09-10-0679

[46] Rosenkranz A, Hiden M, Leschnik B, Weiss EC, Schlembach D, et al (2008) Calibrated automated thrombin generation in normal uncomplicated pregnancy. Thromb Haemost 99: 331–337. 18278182 10.1160/TH07-05-0359

[47] Butenas S, Undas A, Gissel MT, Szuldrzynski K, Zmudka K, et al (2008) Factor XIa and tissue factor activity in patients with coronary artery disease. Thromb Haemost 99: 142–149. 18217146 10.1160/TH07-08-0499

[48] Wulfkuhle KC, Butenas S, Bernstein I, Brummel-Ziedins K (2011) Tissue factor dependent and independent thrombin generation across pregnancy. J Thromb Haemost 9 Suppl 2: 431.

[49] (2012) What happened to personalized medicine? Nat Biotechnol 30: 1. 22231070 10.1038/nbt.2096

